# Management of SARS-CoV-2 Infection: Key Focus in Macrolides Efficacy for COVID-19

**DOI:** 10.3389/fmed.2021.642313

**Published:** 2021-04-14

**Authors:** Gaber El-Saber Batiha, Marwa A. Zayed, Aya A. Awad, Hazem M. Shaheen, Suleiman Mustapha, Oscar Herrera-Calderon, Jorge Pamplona Pagnossa, Abdelazeem M. Algammal, Muhammad Zahoor, Achyut Adhikari, Ishan Pandey, Sara T. Elazab, Kannan R. R. Rengasamy, Natália Cruz-Martins, Helal F. Hetta

**Affiliations:** ^1^Department of Pharmacology and Therapeutics, Faculty of Veterinary Medicine, Damanhour University, Damanhour, Egypt; ^2^Department of Crop Protection, University of Ilorin, Ilorin, Nigeria; ^3^Department of Pharmacology, Bromatology and Toxicology, Faculty of Pharmacy and Biochemistry, Universidad Nacional Mayor de San Marcos, Lima, Peru; ^4^Biological Sciences Department, Federal University of Lavras (UFLA), Lavras, Brazil; ^5^Department of Bacteriology, Immunology, and Mycology, Faculty of Veterinary Medicine, Suez Canal University, Ismailia, Egypt; ^6^Department of Biochemistry, University of Malakand, Chakdara, Pakistan; ^7^Central Department of Chemistry, Tribhuwan University, Kritipur, Nepal; ^8^Department of Pathology, Motilal Nehru Medical College, Prayagraj, India; ^9^Department of Pharmacology, Faculty of Veterinary Medicine, Mansoura University, Mansoura, Egypt; ^10^Green Biotechnologies Research Centre of Excellence, University of Limpopo, Polokwane, South Africa; ^11^Faculty of Medicine, University of Porto, Porto, Portugal; ^12^Institute for Research and Innovation in Health (i3S), University of Porto, Porto, Portugal; ^13^Laboratory of Neuropsychophysiology, Faculty of Psychology and Education Sciences, University of Porto, Porto, Portugal; ^14^Department of Medical Microbiology and Immunology, Faculty of Medicine, Assiut University, Assiut, Egypt

**Keywords:** COVID-19, SARS-CoV-2 infection, macrolides, azithromycin, efficacy

## Abstract

Macrolides (e.g., erythromycin, fidaxomicin, clarithromycin, and azithromycin) are a class of bacteriostatic antibiotics commonly employed in medicine against various gram-positive and atypical bacterial species mostly related to respiratory tract infections, besides they possess anti-inflammatory and immunomodulatory effects. Coronavirus Disease 2019 (COVID-19) is an infectious disease caused by the severe acute respiratory syndrome of coronavirus 2 (SARS-CoV-2). It was first detected in Wuhan, Hubei, China, in December 2019 and resulted in a continuing pandemic. Macrolides have been extensively researched as broad adjunctive therapy for COVID-19 due to its immunostimulant abilities. Among such class of drugs, azithromycin is described as azalide and is well-known for its ability to decrease the production of pro-inflammatory cytokines, including matrix metalloproteinases, tumor necrosis factor-alpha, interleukin (IL)-6, and IL-8. In fact, a report recently published highlighted the effectiveness of combining azithromycin and hydroxychloroquine for COVID-19 treatment. Indeed, it has been underlined that azithromycin quickly prevents SARS-CoV-2 infection by raising the levels of both interferons and interferon-stimulated proteins at the same time which reduces the virus replication and release. In this sense, the current review aims to evaluate the applications of macrolides for the treatment of COVID-19.

## Introduction

Humanity is currently facing a deadly threat, a severe acute respiratory syndrome coronavirus 2 (SARS-CoV-2) pandemic, which is due to the novel 2019 coronavirus outbreak, also known as Coronavirus Disease (COVID-19) (1). As of November 22, 2020, COVID-19 was confirmed in 57,882,183 people worldwide, resulting in the premature deaths of more than 1,377,395 individuals. According to the World Health Organization (WHO), more than 220 countries have reported cases of the deadly virus (2). The continually increasing figures of diagnosed cases and the rise in mortality rate call for immediate, accessible treatment that is effective against the deadly virus (1). As a consequence, the use of macrolides for therapeutic applications is gaining much attention.

Macrolides (for example, erythromycin, fidaxomicin, clarithromycin, and azithromycin) are a class of bacteriostatic antibiotics commonly employed in medical practice against various gram-positive and atypical bacterial species mostly related to respiratory tract infections. Besides their antibacterial properties, macrolides are reported to also have anti-inflammatory and immunomodulatory effects (3–5). The infected host typically links viral respiratory infections, such as COVID-19, to an intense inflammatory response, characterized by hyperproduction of cytokine. Past preclinical and clinical studies have shown that macrolides control the susceptibility to inflammation, increase the buildup of anti-inflammatory cytokines, and also promote the antibody-building cycle. Due to its immunomodulating activities, macrolides have been researched to a great extent as a broad adjunctive therapy against viral respiratory infections, influenza inclusive (6). In this sense, this review seeks to evaluate the applications of macrolides in the possible treatment of COVID-19, with primary considerations on the most relevant macrolide, i.e., azithromycin, in a plausible curative mixture.

## Research Methodology

A MEDLINE literature search was performed using the following keywords: azithromycin and severe acute respiratory syndrome-coronavirus 2 (SARS-CoV2); COVID-19 and azithromycin; viral infections and azithromycin; azithromycin and chloroquine; Qt prolongation; and Qt prolongation and azithromycin. Here, we provided information on the most recent shreds of evidence investigated and on those essential in the synthesis and usage of macrolides as a possible effective treatment of COVID-19.

## Azithromycin: An Overview

### Description

Azithromycin is described as an azalide that is structurally associated with the macrolide family of antibiotics. Macrolides are known to possess immunomodulating and anti-inflammatory activities apart from its antibiotic properties, with such components being able to proffer some effectiveness in a broad range of viral infections respiratory in nature (7).

### Pharmacokinetics

Azithromycin has a bioavailability of ~37%. However, it has been observed that the concomitant oral introduction of azithromycin in combination with food could significantly reduce the drug bioavailability by 50%. Subsequently, a one-time dosage (oral) of 500 mg with a plasma peak concentration within the range of 0.35 to 0.45 mg/L could be reached nearly within 2 h (8). Considering the concentration of 500 mg administered orally on a day followed by 250 mg on days 2 to 5, the mean and peak plasma concentrations would be, respectively, close to 0.25 and 0.05 mg/L. Such low concentrations are due to the effect of substantial and fast spread from plasma to tissues. The binding protein of the associated plasma is considered to be in a small amount of about 50% less at a plasma concentration with the normal treatment dose. The noticeable circulation volume is substantially high at a volume of 25–35 mg/kg (9).

Azithromycin, naturally eradicated by biliary and trans-intestinal excretion discharges, is not involved in stools, with urine excretion being considered a limited route for its removal. Approximately 6% of the oral and 12% of the intravenous doses are recovered without urinary changes. It takes the mean period of about 2–4 days for azithromycin to reach terminal elimination half-life (8). For aged patients, and even in cases of mild-to-moderate kidney or hepatic inadequacy, azithromycin pharmacokinetics is not substantially altered. The azithromycin dose and its dosage regimen agree with marketing authorization for superinfections of acute bronchitis. Indeed, the pharmacology of azithromycin was previously addressed in humans (10), finding that 37% of an individual oral dosage of 500 mg was bio-accessible and able to produce a serum concentration peak of 4 mg/L. Several concentration treatments (i.e., 500 mg in 2 doses with a 12 h separation followed by 500 mg for 5 days, or double doses of 250 mg with a 12 h separation, followed up by a quantity of 250 mg for 9 days) led to a minor serum concentration peak increase. Moreover, the protein serum binding of azithromycin decreased from 0.02 mg/L, which is at 50% to 0.5 mg/L at 12%. The tissue concentrations of this macrolide-type antibiotic were indicated to be amply increased than what is attainable in serum. For example, after administering dual concentrations of 250 mg each, 12 h apart, peak concentrations of azithromycin were stated in tonsil, prostate, and various other tissues (exceeding 3 mg/kg). However, the amount present in tissues weakened, with noticeable half-lives of ~55.2 h in prostate and 76.8 h in tonsil. In fact, the increased concentrations in such associated tissues indicate that the proposed standard treatment doses of 500 mg in a day followed consecutively by 250 mg for 4 days, or 500 mg for 3 days, would result in tissue concentrations above 3 mg/kg. This evidenced as the concentrations in tissues surpassed that of the associated pathogens' minimum inhibitory concentrations (MICs), with treatment doses proving to be effective in antagonizing soft-tissue and/or respiratory tract infections. Also, a one-time 1 g dose was suggested to be effectual in treating various sexually transmitted infections (11).

Antibacterial agents, such as azalide, particularly, azithromycin, were initially shown to possess several pharmacological effects from other antibacterials that are presently being used. As above mentioned, the drug has an approximate bioavailability of 37%, where a quick dispensation accompanies the fast and substantial spread from serum to the intracellular fluid compartments to tissues. Tissue concentrations surpass the concentrations of serum by 100-fold in line with a 1-time dosage of azithromycin (500 mg), with drug concentrations in macrophage assisting in its ability to fight diseases. As referred, high azithromycin amounts are usually observed in prostate, tonsil, lymph nodes, lungs, and liver, with only minor concentrations being noticed in muscles and fat. For example, a 500 mg dose given on the 1st day, followed by 250 mg daily for 2–5 days, has previously been shown to maintain the azithromycin amount at sites where infections could be indicated and continues to be effective for many days even after cessation of administration. At the pharmacokinetic level, data have indicated that azithromycin has various therapeutic applications and effects (12).

### Toxicity

In a study conducted in rats, it was observed that the use of azithromycin leads to its hepatic accumulation and elevated azithromycin demethylase activity (13), due to interactivity with the cytochrome P450 system of liver cells' smooth endoplasmic reticulum. However, any significant clinical connections of the drug with theophylline or warfarin are yet to be detected, so that these agents should be clinically monitored. Furthermore, some macrolides have been observed to weaken digoxin metabolism, and such a relationship might occur with azalides (13).

The administration of cimetidine, along with azithromycin, has not been properly investigated, so that their significant effect on drugs pharmacokinetics needs to be addressed (13). Nevertheless, administering the drugs with some antacids (for example, Maalox) has shown to decrease the plasma concentrations of azithromycin down to 25%, even though there has been no observation in the reduction of absorption level (13).

## Macrolides' Effect Against Viral Infection

**Figure 1:**
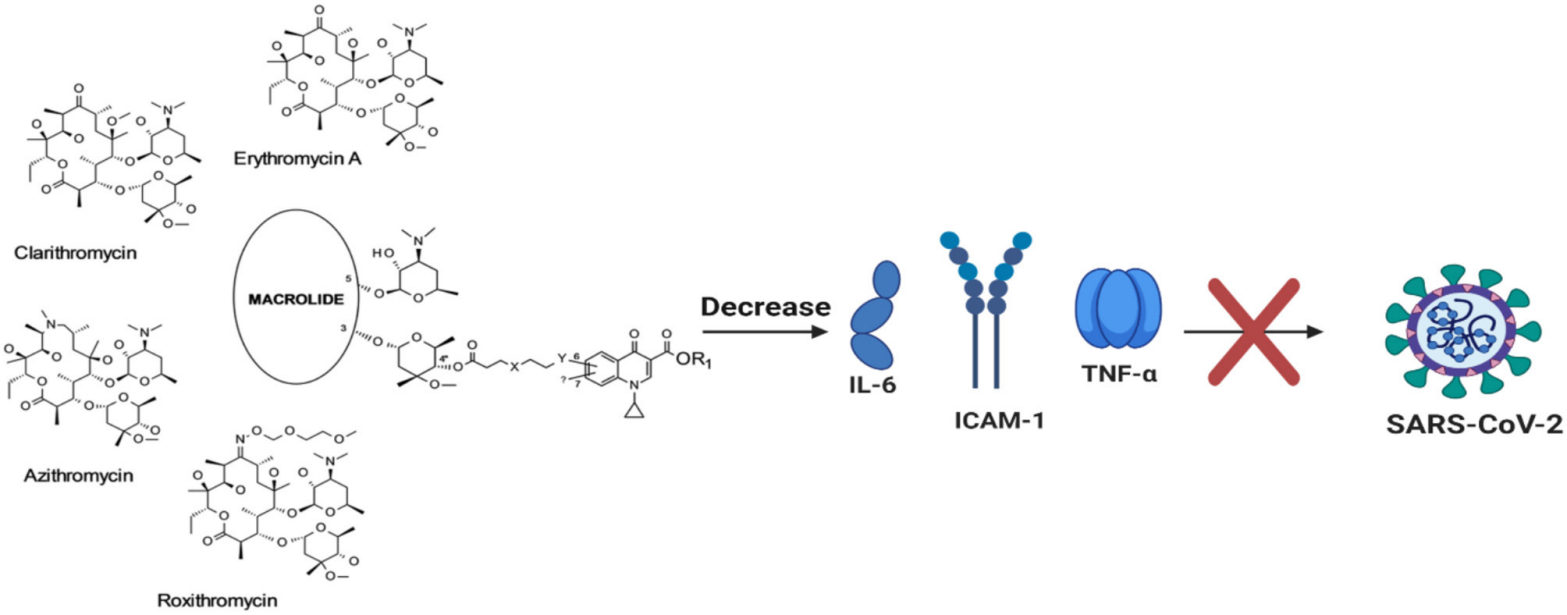
Antiviral effect of macrolides.

In critical cases, the capability of the antibiotic (azithromycin) to decrease the production of matrix metalloproteinases (MMPs), tumor necrosis factor-alpha (TNF-α), interleukin (IL)-6, and IL-8 has been addressed (14). Previously, such macrolide has shown the rise of neutrophil apoptosis and also the oxidative stress linked to inflammation in the resolution phase. Likewise, bafilomycin A1, erythromycin, and clarithromycin were studied to impede the making of IL-1β, IL-6, IL-8, TNF-α, and intercellular adhesion molecule (ICAM)-1 in influenza and rhinovirus-modeled infections (Figure 1) (15–18). To study the potency of bafilomycin A_1_, an inhibitor of vacuolar H^+^-ATPase, on rhinovirus (RV) airway epithelial tissue infections, primary tracheal epithelial cell cultures from human subjects were exposed to type 14 RV infection (RV14). The investigators involved confirmed RV infections by demonstrating that the viral titers in the supernatants of diseased cells and viral ribonucleic acid (RNA) in cells increased over time. The RV14 infection stimulated the cytokine production and also of messenger RNA (mRNA) of ICAM-1 in epithelial cells. Interestingly, bafilomycin A_1_ repressed the ICAM-1 production and decreased the viral titers of RV14 before and after its confirmed infection. Furthermore, the susceptibility of the epithelial cells to RV14 was decreased by the macrolide antibiotic-bafilomycin A_1_.

Observations also revealed that RV14 enhanced the activation of nuclear factor-κappa B (NF-κB) in cells whereby bafilomycin A_1_ lowered it. Also, the number of acidic endosomes present in the epithelial cells was observed to be reduced by bafilomycin A_1_. Such reactions were indicative of the bafilomycin A_1_ potential to hinder RV14 infection by obstructing the RV RNA entrance into endosomes and also by decreasing the ICAM-1 expression in the associated epithelial cells. Thus, the use of bafilomycin A1 could regulate respiratory tract inflammation after RV infection (15). Influenza viruses that affect humans are also linked to the sialic acid α-2,6-galactose sialyloligosaccharides (SAα2,6Gal) present in the respiratory epithelial cells, the uncoating of this virus, and also their passages into the epithelial cells need a low endosome pH level to complete.

In addition, bafilomycin A_1_ is able to restrain the growth of both type A and B influenza viruses in humans as indicated in Madin–Darby canine kidney cells (MDCK). Nevertheless, the repressive potential of this macrolide, clinically used to treat human influenza virus infections in respiratory tract, is yet to be thoroughly investigated. On the other hand, to evaluate the potency of clarithromycin in human influenza virus infection, epithelial cell samples from human windpipes (tracheal) were cultured and then subjected to infection by Influenza A virus subtype H_3_N_2_. The observed infection augmented the cytokine level and also the viral load, as well as IL-1β and IL-6 in supernatant liquids and also in viral RNA within cells. In addition, the cytokine contents in the supernatant liquid, the viral loads, the viral RNA within cells, and the infection vulnerability were generally decreased following clarithromycin use. Also, the expression of sialic acid α-2,6-galactose sialyloligosaccharides (SAα2,6Gal), a receptor for influenza virus in man, was reduced by clarithromycin on the mucous membrane surface of the tracheae. Clarithromycin was also observed to reduce the amount and intensity of acidic endosome fluorescence in cells where viral ribonucleoproteins (RNP) pass into the cytoplasm. Clarithromycin was stated to have, in nuclear extracts, decreased the NF-κB proteins, as also p50 and p65. These outcomes indicate that seasonal human influenza virus could be successfully constrained after clarithromycin use, reducing the sialic acid α2,6-galactose sialyloligosaccharides (SAα2,6Gal), partially inhibiting NF-κB and raising the endosome pH level in respiratory epithelial cells. Thus, viral respiratory tract inflammatory infections could positively be controlled by clarithromycin (18). In addition, some data have also indicated that clarithromycin is able to inhibit IL-6, IL-8, and ICAM-1 and might thus be beneficial on the pathophysiological variations associated with RV infections. For example, the effects of clarithromycin on RV infections in adenocarcinomic human alveolar basal epithelial cells (A549) were thus examined. The procedure used was to treat cells with respect for 1, 10, and 100 μm of clarithromycin, beginning at 3 days before laboratory infections or using clarithromycin at the time of infection. RV titers, according to the measurement by culture on Medical Research Council cell strain 5 (MRC-5), were decreased by clarithromycin, with this decrease being higher when treatment was applied 3 days before infection compared to when it was performed at the time of infection. Besides RV-induced development of IL-1β, IL-6, and IL-8, clarithromycin therapy was found to inhibit RV, with elicited increment in ICAM-1 mRNA and protein. The maximal treatment effect was detected at the post-infection period (after 3 days). Conversely, the IL-8 production is not significantly repressed following clarithromycin administration at the time of infection. Associated findings further suggest that treatment with clarithromycin may hinder the raise in cytokines, the induction of ICAM-1 expression, and viral infection in adenocarcinomic human alveolar basal epithelial cells (A549) (17).

On the other side, Murphy et al. (19) found that azithromycin connected to T-helper (T_H_) phenotype led to a shift in both types one and two, also supporting the repairs of damaged tissues due to inflammation. Furthermore, azithromycin has been reported to attenuate the reactions of endotoxin-lipopolysaccharide on lung allogeneic graft bronchial epithelium cells (20–24). In another study, the use of azithromycin and midecamycin in respiratory epithelial cells of many human patients suppressed the raise in mucin and TNF-α levels (24). Indeed, it was stated that midecamycin and azithromycin lessened the phorbol myristate acetate (PMA)-induced MUC2 and MUC5AC mucin gene and protein expression in NCI-H292 cells while repressing the phorbol myristate acetate-mediated TNF-α. Also, azithromycin was revealed to be effective in reducing biofilm formation and in restraining the quorum sensing and bacterial protein synthesis. Effective azithromycin aggregation in cells, mostly phagocytes, is usually carried over places with infections, as expressed in the extensive distribution of tissue and plasma clearance. Additionally, azithromycin has been recommended for the treatment of bacterial infections, including dermal, genitourinary, and respiratory infections. It has also shown immunoregulatory efficacy in persistent and inflammatory diseases, including rosacea, diffuse panbronchiolitis (DPB), and transplant-related complications, including post-transplant bronchiolitis. Host-response modulation accelerates the long-lasting medicinal effectiveness in non-eosinophilic asthma (NEA), chronic obstructive pulmonary disease (COPD) exacerbations, and both cystic and non-cystic fibrosis bronchiectasis. Azithromycin also exerts stimulative activities on epithelial and immune cells linked to extracellular signal-regulated kinases (ERK) 1 and 2 and phospholipids, accompanied by the late modulation of NF-κB and activator protein-1 (AP-1) transcription factor, mucin release, and cytokine-related inflammation (25). It also exerts late repressive effects on cells' smooth function and also increase lysosomal buildup as a consequence of the disturbance in both intracellular lipid and protein transportation, while also exerting surface receptor expression modulation of autophagy and macrophage phenotype. Such alterations underlie several immunomodulatory activities, adding to the timely resolution of severe infections while mitigating aggravations in chronic respiratory tract diseases. Previously analyzed were groups of people with post-transplant bronchiolitis, who revealed to be responsive to azithromycin treatment, as well as some people with acute sepsis (26), periodontitis, and prostatitis; notwithstanding, a weakened effect is not likely to be critical in malaria. Anyway, given data currently available, the continual application of azithromycin needs to be well-adjusted against the possibility of bacterial resistivity. Indeed, despite that some reports have stated rare situations of torsades de pointes (TDP) in people who are at risk, the use of azithromycin has continued to show an encouraging track record of safety in its usage (23). An experiment was performed to study the function of human neutrophils and to circulate inflammatory mediators on 12 volunteers each receiving a different dose of 500 mg of azithromycin per day (p.o.) for 3 days (20). Blood was drawn from volunteers in the following order: 1 h before treatment, 2 and a half h, 24 h, and also 28 days after administration. Neutrophil-degranulating activities following azithromycin use were stated to quickly decrease the activities of azurophilic granule enzymes in cells and to increase their serum level. Also, oxidation responses to the particulate stimulus were indicated to be highly improved. The activities observed were connected to an increase in neutrophil and plasma drug concentrations (20). The constant decrease in IL-6 serum and chemokine concentrations within ranges that were non-pathological followed a rise in apoptosis, and a slowing downregulation of the oxidative burst of neutrophils up to 28 days after administration of the final treatment dosage of azithromycin was also reported. The isolated blood neutrophils at this time still had identifiable amounts of the drug. Indeed, severe neutrophil stimulation may positively enhance azithromycin microbicidal effectiveness while the slowing down of possibly anti-inflammatory activities could restrain detrimental inflammations (20).

On the other hand, most asthma aggravations are due to RV infections (27). Currently available therapy for such exacerbation is believed to be insufficient. Preceding indications suggested that macrolide antibacteria possess both antiviral and anti-inflammatory capabilities even though the procedure directly involved is yet to be known. Furthermore, a study was carried out to investigate the anti-RV effects of macrolides (telithromycin, erythromycin, and azithromycin) by the induction of protein and antiviral messenger RNA (mRNA) in primary human bronchial epithelial cells (HBEpC), subjected to major group RV-6 and minor group RV-1B infection. The antiviral gene mRNA expression, IL-6 and IL-8 levels, RV release and replications, interferon-λ2/3, type I interferon-β and type III interferon-λ1, and interferon-stimulated genes (oligoadenylate synthase, melanoma differentiation-associated gene 5, retinoic acid-inducible gene I, viperin, and MxA) were carefully addressed (22). Azithromycin treatment led to a significant increase in protein production and interferon and gene mRNA expression enhanced by RV-1B and RV-16, whereby erythromycin and telithromycin treatments were not significant enough. Moreover, azithromycin treatment further decreased the release of RV and its replication significantly. Observation also revealed that the RV-stimulated IL-6 and IL-8 protein and mRNA expressions were not indicated to be significantly decreased with azithromycin use (22). Indeed, there is a lot of evidence underlining that azithromycin has good anti-inflammatory potency (28). For instance, in acquired and innate immunity, macrophages are essential and enhance the pro-inflammatory cytokine production, such as IL-12, which comprises both p35 and p40 subunits. The primary aim of IL-12 is to boost type 1 T-helper (T_H_1) production and response. Thus, the effect of azithromycin on IL-12 p40 production in macrophages following stimulation of interferon-γ and lipopolysaccharide were investigated. Azithromycin was used to pretreat the RAW264.7 macrophage cell line, which was followed by the production of lipopolysaccharide and interferon-γ (21). Real-time polymerase chain reaction (RT-PCR) and enzyme-linked immunosorbent assay (ELISA) were employed to evaluate the IL-12 production. Furthermore, reporter assay and electrophoretic mobility shift assay were also used to assess the IL-12 transcriptional regulation (29). Interferon consensus sequence-binding protein (ICSBP) and phosphorylation of activator protein-1 were evaluated by both immunoblotting (using specified antibodies against ICSBP and Jun-B) and immunoprecipitation (using phosphotyrosine), whereby azithromycin application decreased the induction of the IL-12 p40 promoter by lipopolysaccharide/interferon-ÿ in a dosage-dependent way. The nuclear factor-mediated T-cells, activator protein-1, and interferon consensus sequence-binding protein to the DNA-binding site in the IL-12 p40 promoter were also indicated as being inhibited by azithromycin (21). Observations further revealed that azithromycin minimized the activities of the lipopolysaccharide/interferon-γ-induced IL-12 p40 promoter. Stimulated cells that had been treated with azithromycin inhibited both JunB and ICSBP phosphorylation. Finally, the activities of IL-12 p40 transcription were decreased by azithromycin through inhibition of AP-1, ICSBP, and nuclear factor-activated T-cells in the promoter site. Such could be representative of a key procedure for the regulation of azithromycin anti-inflammatory activities in macrophages (21).

## Azithromycin Effectiveness in COVID-19 Management

In a France-based clinical study, hydroxychloroquine was administered to 20 patients and compared to 16 patients who served as controls (not treated with hydroxychloroquine). Six patients were given a mixture of 200 mg of hydroxychloroquine administered three times a day for a period of 10 days and 500 mg of azithromycin on the first day (30). The investigating scientists concluded that 100% of patients administered the mixture were virologically attenuated during the 6th day as opposed to about 57.1% of patients who were treated with only hydroxychloroquine and 12.5% of those in the control groups (30). Contrary to this striking finding, in another study, 11 patients received a mixture of azithromycin and hydroxychloroquine [using the same dose described by Gautret et al. (30) and Molina et al. (31)]. It is also interesting to note that in the stated instance by Molina et al. (31), the patients incurred comorbidities significantly, related to conditions that were poor such as hematological cancer, human immunodeficiency virus infection, and obesity. Treatment had to be stopped for one patient just after 4 days due to Qt prolongation. Furthermore, on an updated report, Gautret et al. (30) stated a promising situation (which denotes patients that were no more given treatment and also required no oxygen treatment) in which out of 80 patients (81.3%) observed, 65 of them treated with azithromycin and hydroxychloroquine had a negative viral load test at 6 days in 83% of those treated with the mixture. Fifteen percent needed oxygen treatment, three patients required intensive care unit admission but later got better, while unfortunately, one patient died (30).

Two critical studies on the effectiveness of combining azithromycin and hydroxychloroquine as a treatment combination for the deadly virus were recently published. Rosenberg et al. (32) described a retrospective multicenter cohort analysis on one thousand four hundred and thirty-eight patients with COVID-19 which were hospitalized. As a possible treatment for the virus, 735 patients received azithromycin and hydroxychloroquine. In a combined therapy (azithromycin + hydroxychloroquine), no significant variations were stated when compared to control patients (31). In addition, Mehra et al. (33) stated a situation in contradiction of the advantages of applying the treatment combination of chloroquine or hydroxychloroquine with clarithromycin or azithromycin to a total number of 96,032 COVID-19 patients. Having compared the patients' mortalities (in-hospital) that were given the mixture of quinoline and macrolide derivatives with those who received no treatment for the virus, the authors detected that the treatment combinations were linked to a high risk of death (32, 33). Presently, various trials are still ongoing in evaluating the potency of azithromycin in COVID-19 treatment. Placebo against azithromycin, in mixed form or against hydroxychloroquine or in triple combination with tocilizumab, is the most usual pattern studied (such as NCT04341870, NCT04329832, NCT04334382, NCT04332107, NCT04348474, NCT04341207, NCT04329572, NCT04339426, NCT04336332, NCT04335552, NCT04332094, NCT04339816, NCT04328272, NCT04338698, NCT04347512, NCT04345861, NCT04349592, NCT04321278, NCT04322396, NCT04344444, NCT04322123, NCT04334512, NCT04324463, NCT04351919, NCT04345419, NCT04341727, NCT04332835, NCT04349410, NCT04347031). The other research conducted by a French group also aimed to assess the efficacy, in particular by medical and other health workers who associate patients with the virus and those exposed to it (NCT 04344379) as well as to those at high risk of contracting the virus, using azithromycin and hydroxychloroquine as a way to prevent SARS-CoV-2 infection. The macrolide azithromycin is considered to be one of the drugs that have been included in the large adaptive RECOVERY test and also in the United Kingdom Medicines and Healthcare products Regulatory Agency study jointly sponsored by the University of Oxford with the European Union Clinical Trial (EudraCT) number: 2020-001113-21. In previous *in vitro* screen tests by the Food and Drug Administration (FDA)-approved chemical libraries, azithromycin has been shown to have the half-maximal inhibitory concentration (IC_50_) of 2.12 μM in antagonism to SARS-CoV-2 (34). In connection to the aforementioned inflammatory reactions and effects, macrolides could plausibly exert a prophylactical function in staphylococcal and pneumococcal difficulties that come on with a particular incidence as complications of respiratory tract viral infection.

## Cluster of Differentiation 147 (CD147) and COVID-2019 Treatment: Possible Effects of Azithromycin and Stem Cell Engagement

**Figure 2:**
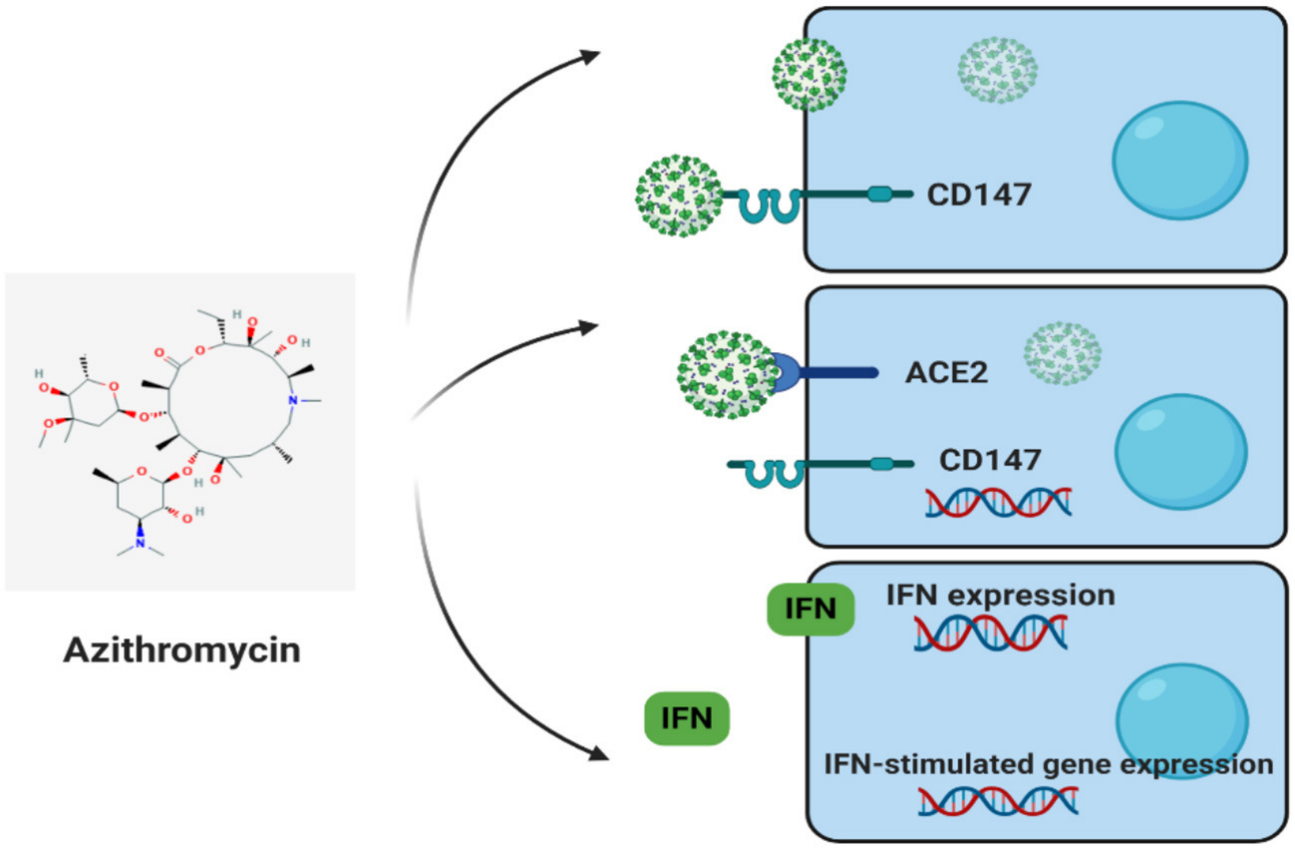
Targets and conceivable treatments for coronavirus disease-2019.

Angiotensin-converting enzyme 2 (ACE2) and cluster differentiation 147 (CD147) (also known as EMMPRIN or Basigin) are host cell receptors and novel routes for SARS-CoV-2 invasion (Figure 2) (35). In host cells, ACE2 or CD147 binds to virus spike protein (S-protein), disseminating virus, and mediating viral invasion within other cells. The SARS-CoV-2-S-protein is similar to SARS-CoV S-protein, with both binding to ACE2, attacking host cells (35). Apart from ACE2, Wang et al. recently showed that CD147 could also bind to SARS-CoV-2-S-protein (35). Their investigation employed the use of an anti-CD147-Meplazumab, co-immunoprecipitation, ELISA, and immuno-electron microscopy to reveal the new CD147-S-protein route of viral invasion. As main statements, the study underlined a significant focus on the application and development of specified anti-SARS-CoV-2 medications. Furthermore, another medical trial by the Chinese in its second phase with the title: “Clinical Study of Anti-Cluster of Differentiation 147 Humanized Meplazumab for Injection to Treat with 2019-nCoV Pneumonia” (ClinicalTrials.gov Identifier: NCT04275245) is presently being conducted with the chief aim of restricting the CD147 protein by monoclonal antibodies to impede SARS-CoV-2-S-protein binding and further infections. The experiment is currently supervised by virologic clearance rate by using a RT-PCR of airway tract samples to evaluate the activities of Meplazumab. The drug is viewed as a very exact molecule against CD147. Despite such a distinction, it is not exempted from the facts that other medication affecting CD147 expression could also possess advantageous effectiveness in the treatment of COVID-19.

Also worth noting is that CD147 acts as a receptor for *Plasmodium falciparum* (protozoan that causes Malaria) invasion on red blood cells (RBCs). CD147, in addition to ACE2, also acts as a host cell receptor for SARS-CoV-2 invasion. Indeed, host cell invasion by SARS-CoV-2 (*in vitro* and phase II clinical trial) and *P. falciparum* is prevented by using treatment with anti-CD147. Azithromycin also quickly prevents SARS-CoV-2 infection and *P. falciparum* invasion, probably interfering with vital ligand/receptor interactions. However, azithromycin effectiveness in SARS-CoV-2 is soon to be assessed. As a matter of fact, antiviral responses in epithelial cells are induced by azithromycin by raising the levels of interferons and interferon-stimulated proteins and also reducing the virus replication and release. In addition, it also reduces MMP expression, a molecule nearly associated with CD147. *In vitro*, high glucose levels and influenza A virus in cells of patients with asthma increase CD147 expression, thus indicating plausible relationships with diabetes mellitus, asthma, and CD147 levels in medical complications due to SARS-CoV-2 infection.

## Hydroxychloroquine and Azithromycin on SARS-CoV-2 Infection: Recent Trends

As referred above, azithromycin is a well-known drug and considered to be generally safe. In the United States of America, it has been extensively prescribed, e.g., with 12 million azithromycin prescriptions dispensed to children under 19 years old (36). Recent investigations placed both hydroxychloroquine and azithromycin as among 97 possible effective drugs for COVID-19 management (37). In a preliminary clinical study, the use of hydroxychloroquine and/or with the combination of azithromycin to boost the drug potency was discovered to have positive effects in the mitigation of SARS-CoV-2 viral load in certain infected patients (30). Since the start of the worldwide pandemic, numerous strains have been isolated, of which one was SARS-CoV-2 IHUMI-3 which was examined by mixing different concentrations of both hydroxychloroquine and azithromycin with Vero E6 cells. Chloroquine has a reputation for both its immunomodulatory and anti-malaria effects and is also widely accepted and used worldwide (38–42). A new study (*in vitro*) indicated that the use of chloroquine inhibited the growth rate of SARS-CoV-2 (43). Such discovery is in line with other investigations conducted in ~100 admitted people with the virus (44, 45).

An analog of chloroquine is hydroxychloroquine, which has little concern on the drug-to-drug interactions. In an earlier SARS-CoV outbreak, hydroxychloroquine has been described to possess anti-SARS-CoV action in *in vitro* investigations (46). This indicates the possible pharmacological role that hydroxychloroquine could play in COVID-19 treatment and management. However, there is currently no medical proof to support the drug administration as a confirmed cure for SARS-CoV-2 infection. Nonetheless, the mechanism of action of both hydroxychloroquine and chloroquine molecules has not been extensively studied, as previous investigations had only hinted on the drug inhibitory potential through a sequence of steps against coronavirus. The treatments could alter the pH level of cell membrane surfaces and therefore could restrain the viral union to the cell membrane. Furthermore, this treatment could also hinder the following: the replication of nucleic acid, virus assembly, glycosylation of viral proteins, new virus particle transport, virus release, and other processes to attain antiviral reaction (47).

A consistent and dependable estimate of the amount of both chloroquine and hydroxychloroquine in lungs and other related tissues could thus be employed for treatment doses recommendation. A physiologically based pharmacokinetic (PBPK) model may be depended on for this estimate. PBPK is a technique that uses mathematical modeling to predict the drugs' concentration in tissues of humans *in silico* by integrating physiological and drug disposition parameters. PBPK are extensively required for the development of medicine in order to assist in identifying if a clinical trial is justified and validated in addition to aiding guidance on the use of such drugs based on appropriate authorized prediction models (48, 49).

Despite the efforts of innumerous researchers focusing on finding a therapeutic agent targeting directly SARS-CoV-2, a very incipient progress has been achieved in identifying curative therapies. By October 2020, many of the potential drugs/vaccines still figured as possible COVID-19 therapeutic agents and extensive clinical studies were warranted to verify their safety and efficacy (50). Regarding that containing the spread of the infection is the best way to control the COVID-19 pandemic, a vaccine that prevents future infections must be considered as a key priority. Also, the prevention of contamination and identification of clinical symptoms via diagnostics that rapidly and accurately detect SARS-CoV-2 infection are extremely important (51).

Hence, vaccines are the primary and most promising solution to mitigate new viral strains. The genome sequence and protein structure of SARS-CoV-2 were made accessible in record time, allowing the production of inactivated or attenuated viral vaccines. Nanotechnology also improves modern vaccines since they are ideal for antigen delivery, as adjuvants, and as mimics of viral structures (52). According to Iyer et al. (53), containing the spread of SARS-CoV-2 infection will potentially reduce the stress and pressure on the health-care professionals, as well as on the researchers/scientists, capacitating them to deliver better effort on the development of specific vaccines for COVID-19.

## Azithromycin, Hydroxychloroquine, and Chloroquine: Potentially Unfavorable Outcomes

Besides the usual unpleasant effects, like headache, nausea, and itching, the utilization of hydroxychloroquine and chloroquine could lead some patients to severe arrhythmias, a reaction that the concomitant use of azithromycin could facilitate. Some severe but unusual possible dangers that could also occur comprise drug-to-drug interactions, idiosyncratic hypersensitivity reactions, neuropsychiatric effects, and hypoglycemia, with genetic variability which might also have a vital part in them. Not to mention, hydroxychloroquine and chloroquine are highly toxic when taken in excessive amounts (54).

### QTc Interval Prolongation

It is important to note that hydroxychloroquine and chloroquine interfere with ventricular repolarization, which leads to QTc interval prolongation and also heightened threats of torsades de pointes (TdP). This reaction is reliant on the kind of concentration of drug administered. Previous investigations observed that the mean QTc level of some volunteers increased to about 6.1 ms after administering a concentrated dose of 600 mg, and 28 ms after a dose of 1,200 mg (55, 56). Notwithstanding, such effects differed from person-to-person and were usually pronounced. Out of 30 children who received small concentrations of chloroquine to treat malaria, 1 had a rise of about 64 ms in QTc interval 1 day after taking the drug (57). The use of azithromycin alone is not known to significantly cause any QTc interval prolongation in clinical trials (58); however, in theory, when azithromycin is used together with hydroxychloroquine or with chloroquine, it may increase the TdP threat. Interestingly, no indication of such risk was reported when an animal model was used (59). Furthermore, the combined drugs have been safely and successfully used in the treatment of patients with malaria (60, 61). Nonetheless, with not much experience in treating patients with COVID-2019 and also the possible use of these medicines to treat cardiac disease or patients under the influence of drugs that delay repolarization, it is highly recommended to daily monitor the QTc interval at baseline throughout treatment, particularly if azithromycin has been prescribed. During prophylactic treatment, day-to-day monitoring may not be feasible; however, the baseline assessment of the QTc interval is highly recommended, specifically for patients diagnosed with cardiac disease. Where possible, it is well-advised to treat any electrolyte disorders and to limit or avoid the use of any medication with the ability to prolong the QT interval.

### Microbiota Affectation With Macrolides

Regarding the difficulty of differentiating COVID-19 from bacterial pneumonia as stated by clinicians (62), many considerations about the use of antibiotics should be taken such as the following: the uncertainty of the presence of bacterial superinfections; the absence of specific antiviral agents with proven efficacy; the relative high mortality; and the antimicrobial resistance caused by selective pressure on natural microbiota of individuals. The natural microbiota reduction can be considered a serious concern especially for individuals suffering mild symptoms of COVID-19. Still, even with the negative side effects, antibiotics should be considered as an alternative for treatment method for the most severe suspected or confirmed COVID-19 cases (e.g., patients presenting hypoxic respiratory failure demanding mechanical ventilation) (63).

Stricker and Fesler (64) state in their study that treatments using antibiotics somehow protect patients against SARS-CoV-2 and severe COVID-19 disease. Besides, in normal times, the medical practices do not recommend using antibacterial medications to treat viral infections in order to avoid overuse of antibiotics. Yet, the arrival of SARS-CoV-2 has forced a major review in the extensive medical literature in which some studies demonstrate potential antiviral and anti-inflammatory effects of numerous antibacterial agents such as azithromycin (65), ciprofloxacin (66), spiramycin (67), clarithromycin (68), nitazoxanide (69), metronidazole (70), doxycycline (71, 72), and other tetracyclines (73).

## Azithromycin for COVID-19 Management: A Further Consideration for Safe Use

A recent report by Juurlink (54) on hydroxychloroquine, chloroquine, and azithromycin did not address a safety concern in azithromycin application, which is imperative to recipients of allogeneic blood and marrow transplantation, in which the use of azithromycin could be advised for treating or preventing transplant-related lung diseases (i.e., bronchiolitis obliterans) (54). In a 2017 controlled randomized study involving participants who were receiving blood and marrow transplant, individuals assigned to receive prophylactic azithromycin were found to have significantly higher relapse rates for their underlying hematologic malignant disease, in addition to a rise in death rate generally (74).

## Conclusion

Azithromycin is a well-known antibiotic with good anti-inflammatory and immunomodulatory effects and considered to be generally safe, besides being reported as useful for the treatment of COVID-19. The combination treatment of hydroxychloroquine and azithromycin was documented to have positive effects to mitigate SARS-CoV-2 viral load in certain patients. Azithromycin and other macrolides, like bafilomycin A1, erythromycin, and clarithromycin were found to impede the making of IL-1β, IL-6, IL-8, TNF-α, and ICAM-1 in influenza- and RV-modeled infections.

## Author Contributions

All authors listed have made a substantial, direct and intellectual contribution to the work, and approved it for publication.

## Conflict of Interest

The authors declare that the research was conducted in the absence of any commercial or financial relationships that could be construed as a potential conflict of interest.
